# Analysis of Refractive Errors in a Large Italian Cohort of Pediatric Subjects Post the COVID-19 Pandemic

**DOI:** 10.3390/life13071569

**Published:** 2023-07-15

**Authors:** Michele Lanza, Adriano Ruggiero, Matteo Ruggiero, Clemente Maria Iodice, Francesca Simonelli

**Affiliations:** Multidisciplinary Department of Medical Surgical and Dental Specialties, University of Campania Luigi Vanvitelli, 80138 Naples, Italy

**Keywords:** astigmatism, children, COVID-19, eyecare, hypermetropia, myopia, pandemic, prevalence, refractive errors

## Abstract

Background: The prevalence of refractive errors has sharply risen over recent decades. Despite the established role of genetics in the onset and progression of such conditions, the environment was also shown to play a pivotal role. Indeed, the COVID-19 pandemic has majorly impacted people’s lifestyles and healthy habits, especially among the youth, which might have led to a significant increase in this trend. Therefore, the aim of this study was to investigate the actual prevalence of refractive errors in a large cohort of pediatric patients. Methods: A large cohort of 496 participants was screened through anamnesis, a non-cycloplegic autorefractometry, a corrected and uncorrected visual acuity assessment, and a questionnaire and was retrospectively evaluated. Results: Overall, refractive errors were present in 25.1% of eyes, of which 14.6% were diagnosed with myopia/myopic astigmatism and 10.5% with hyperopia/hyperopic astigmatism. Among the patients enrolled, 298 (60%) had their eyes checked one year earlier or before and 122 (25%) had never had ophthalmological consultations; a total of 105 (21%) needed glasses and 34 (7%) required a change in their previous prescription. A substantial increase in daily electronic device screen exposure was declared by 426 patients (87.6%). Conclusions: Pediatric patients appear to have a higher prevalence of refractive errors than before.

## 1. Introduction

During recent decades, several cross-sectional studies from all over the world have reported a dramatic rise in the prevalence of refractive errors within the general and, in particular, the young population [1,2,3]. Indeed, far earlier than the COVID-19 pandemic outbreak, there was already widespread concern regarding an emerging, serious youth-related vision impairment worldwide. To raise public awareness, the World Health Organization diffused public reports detailing that about 2.6 billion people globally suffered from impaired vision, among which a significant proportion was made up of individuals under 18 years of age [4]. In addition, it is actually estimated that in about 25 years from now, almost 5.7 billion people, or 59.6% of the total world’s population, will become myopic, allegedly affecting close to one-half of the population worldwide [5]. Moreover, the incidence of high myopia (or near-sightedness) is predicted to rise as well, reaching pre-announced peaks equal to 10% of the global population [3]. In light of this, even though myopia is commonly considered a benign condition, it is important to highlight that severe stages of this disease are strictly associated with demonstrated higher risks of serious vision-threating pathologies, such as macular degeneration, posterior staphyloma, retinal detachment, cataract, and glaucoma [6].

With regards to the relatively recent COVID-19 pandemic, governments worldwide have been required to take unprecedented public health measures [4]. Indeed, during the peak months of the pandemic, 192 countries/territories opted to restrict access or even to close schools as part of a series of preventive maneuvers aimed at restraining inter-individual contacts and thus at reducing the extent of the pandemic’s spread [7]. Such strictly preventive actions resulted in affecting nearly 1.5 billion children and young people’s habits and lifestyles, leading to a heavily increased sedentary time, time spent in near- and intermediate-sight activities (such as reading and table and/or electronic devices use), and an overall insufficient time spent outdoors in physical activities [8].

Consequently, in consideration of the school-aged individuals’ safety and health, a digital learning policy became imperative to reduce the adverse impacts of school closures. However, whilst a combination of remote learning and digital technology offered a timely and efficient solution to the issue, emerging research claimed that the prolonged exposure to electronic devices and digital screens during the COVID-19 pandemic would potentially negatively affect the vision development in pediatric and adolescent individuals [9].

It is important to highlight that the school age is reported to represent a period characterized by intensive eye use, which is pivotal for correct sensory function development [3]. In addition, even though ethnicity was demonstrated to heavily influence the onset and progression of this condition, the environment was shown to play a crucial role as well [3]. Indeed, several studies have demonstrated that myopia is strictly associated with individual traits, being strikingly more prevalent among females than males, among older children, and among urban household individuals [10,11]. On the other hand, recent published literature has widely described that myopic onset and progression could be affected by environmental and lifestyle factors, among which the prolonged, proximal digital screen application was shown to play a pivotal role [12]. Moreover, unbalanced diet regimens and irregular sleep patterns, as well as the increased exposure time to other common close-work activities (e.g., reading and writing), heavily contributed to intensify the incidence and progression of refractive errors, especially within children and adolescents [13,14,15,16].

Some experts have even labeled the condition “quarantine myopia”, where the cumulative effect of such multiple factors have resulted in the rapid increase in the prevalence of myopic refractive errors witnessed over the last few years [8,16,17]. Indeed, among the complex mechanisms of visual feedback modulating eye growth shown to possibly mediate the relationship between outdoor activities and refractive errors, exposure to brightness and chromatic spectra of light, energy at high spatial frequencies, peripheral defocus, and circadian rhythms seem to have a crucial role [15].

Moreover, the overwhelming effects that COVID-19 had on medical facilities worldwide resulted in affecting patients’ routine access to general healthcare, especially in suburban areas where medical care access was limited even before pandemic, and in particular for eyecare, often preventing the prompt diagnosis and management of ocular conditions [18,19]. All the uncorrected ametropias would represent a major cause of visual disability in children and, more importantly, the demonstrated increased incidence of high myopia within the current pediatric population worldwide would be a serious risk factor for potentially sight-threatening complications in the next elderly generations [16].

Therefore, the aim of this study is to investigate the actual incidence of refractive errors in a large cohort of Italian pediatric patients living in a sub-urban area of one of the largest cities in Italy after the COVID-19 pandemic and to offer the community reliable evidence-based data.

## 2. Materials and Methods

### Patients

**Figure 1:**
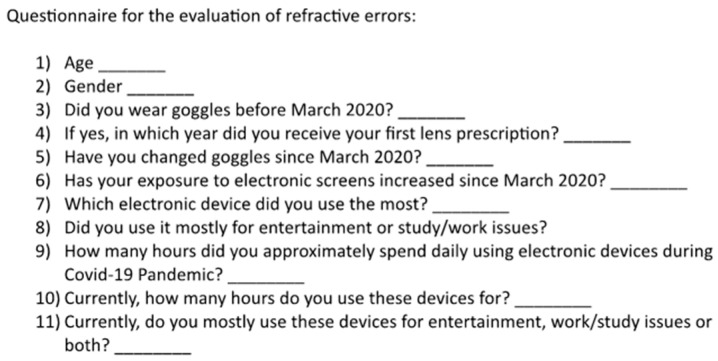
Questionnaire for the evaluation of refractive errors and digital screen exposure.

In this retrospective, cross-sectional, observational study, 988 eyes of 494 children were included. The cohort included 271 males (54.6%) and 223 females (45.4%) with a mean age of 7.82 years (±1.23 years, range 6–11 years). All the enrolled individuals belonged to the same school in a sub-urban area of Naples (Italy), characterized by a substantial lack of dedicated facilities to perform a complete eye visit. After obtaining permission from the school and either the parents or the legal guardians, every student was invited to attend a screening program for early detection of visual conditions during the month of September 2022. Two ophthalmologists (ML and MR) evaluated subjective refraction and ocular motility and administered a questionnaire (Figure 1) to each individual included. All the data were then retrospectively reviewed by two independent clinicians (AR and CMI).

The approval of the study was provided by the local Ethics Committee of the University of Campania Luigi Vanvitelli and was conducted in accordance with both the Helsinki Declaration and the Good Clinical Practice guidelines. Informed written consent was obtained from all parents and subjects prior to study inclusion. The screening tests consisted of anamnesis, a check of the eventual documentation regarding previous visits, a non-cycloplegic autorefractometry, a corrected and uncorrected visual acuity assessment, and a questionnaire. All the participants were asked about their gender and age, and more importantly, were asked about whether they wore glasses before the COVID-19 pandemic outbreak, at which age they received their first prescription, and if they needed to change their glasses refraction ever since. In addition, the daily use of electronic devices and screens was reviewed.

Visual acuity testing was carried out using a standard 3 m retro illuminated decimal Snellen chart (GIMA spa, Milan, Italy) under mesopic lighting conditions. Autorefractometry was carried out in non-cycloplegic conditions, using a Canon RK-F1 device (Canon USA Inc., Lake Success, NY, USA). The administered questionnaire was made up of several questions regarding the change in personal habits that could relate to the onset of vision problems. Continuous variables were reported as a mean ± standard deviation (SD) and categorical features were reported as a count (frequency). Gender differences were explored using the Mann–Whiney U Test, with the statistical significance set at *p*-value < 0.05.

## 3. Results

**Figure 2:**
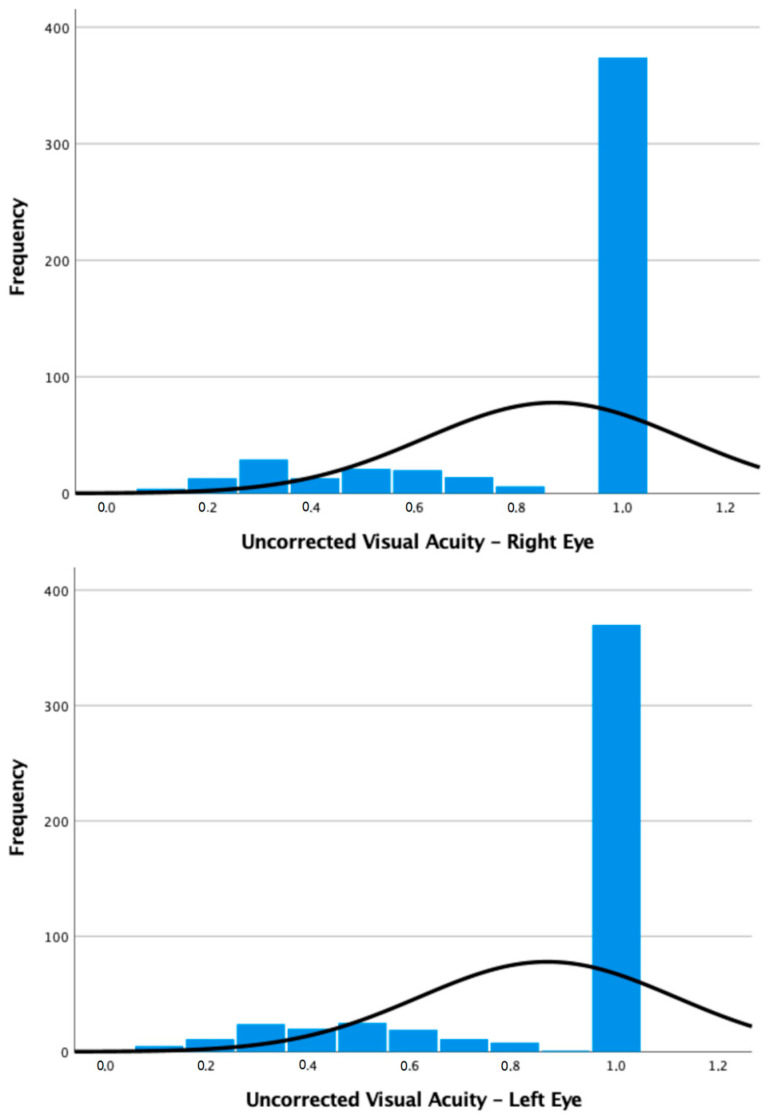
Distribution of UVA by eye. As the right-skewed curve shows, most eyes display the optimal UVA. However, overall, a high percentage (25.1%) of patients was still found to have an impaired UVA and thus needed a lens prescription.

The data of 494 children (988 eyes) were analyzed. The mean uncorrected visual acuity (UVA) was 0.87 decimals (±0.25 decimals) in the right eye (RE) and 0.86 (±0.25 decimals) in the left eye (LE). The mean BCVA was 0.99 decimals in the RE (±0.05 decimals) and 1.00 decimals (±0.00 decimals) in the LE. In the overall cohort, most eyes (74.9%) displayed an optimal UVA of 1.00 decimals (Figure 2). Refractive errors were present in 25.1% of eyes and, in particular, 14.6% were diagnosed with myopia/myopic astigmatism and 10.5% with hyperopia/hyperopic astigmatism. The mean spherical equivalent was −0.06 decimals (±1.44 decimals). Only seven eyes (0.7%) were found to have ocular motility disorders.

**Figure 3:**
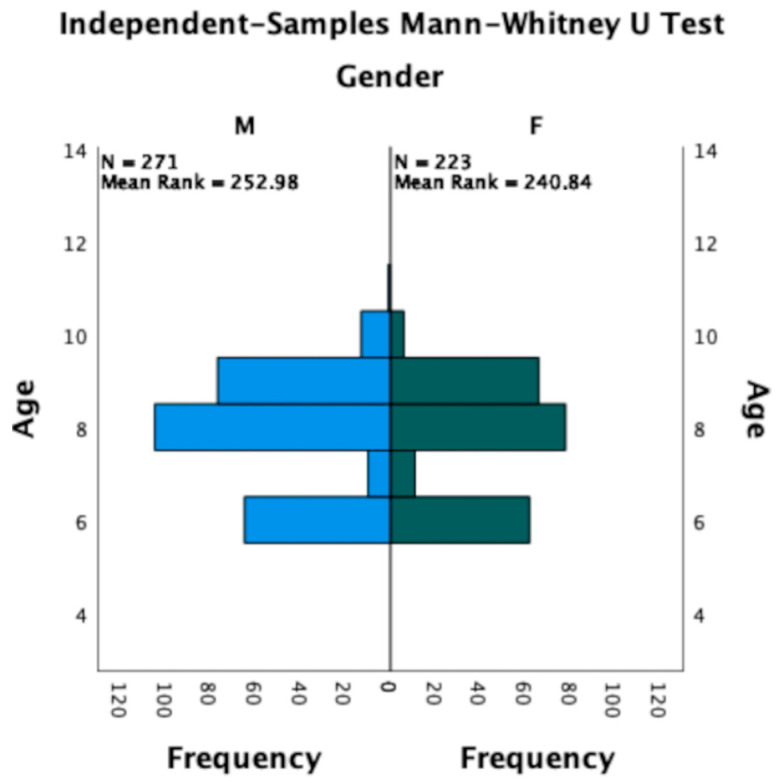
Cohort distribution based on age and gender. No statistically significant difference was found.

The Mann–Whitney U Test showed no statistically significant differences related to age, gender (Figure 3), UVA, and BCVA for each eye, separately.

**Figure 4:**
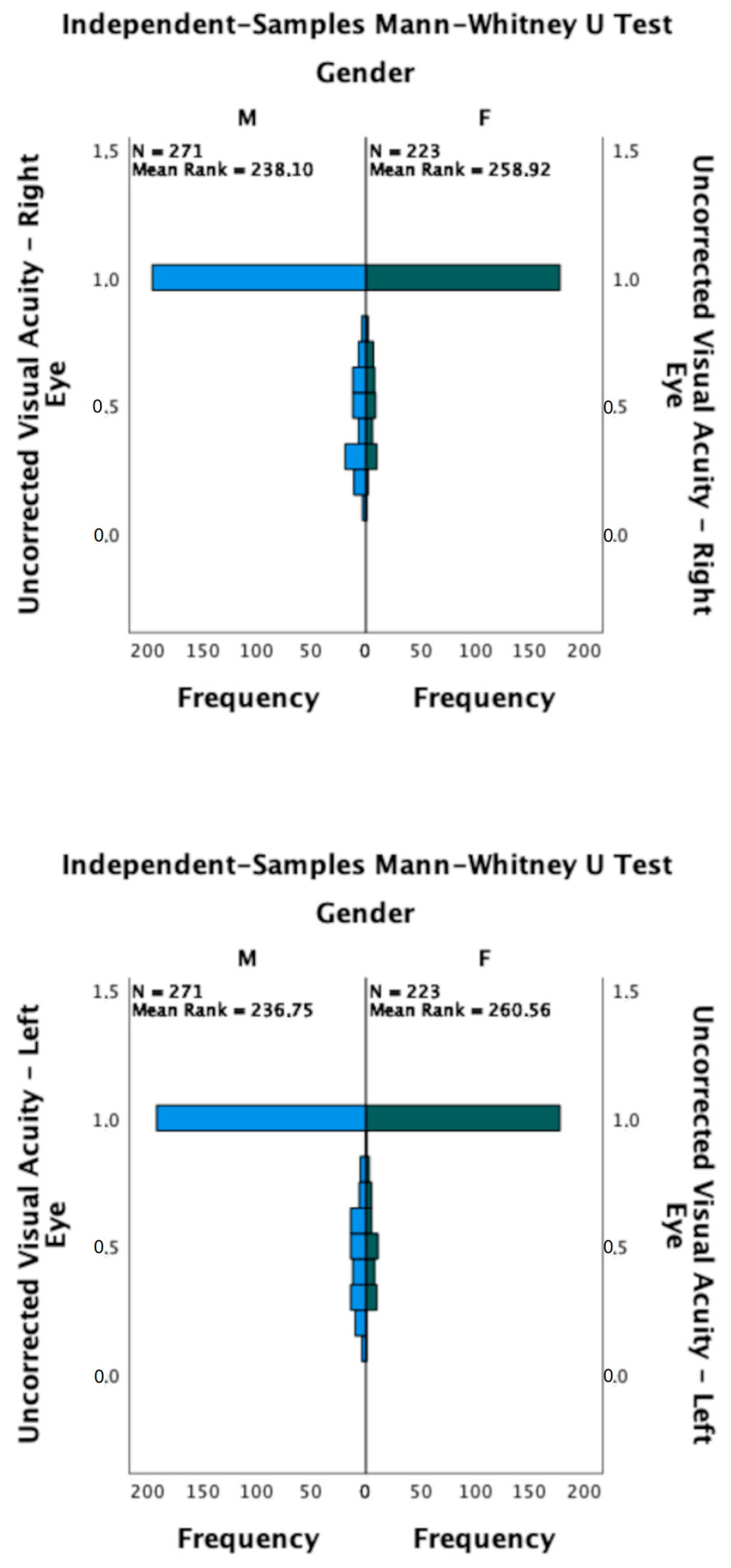
Cohort distribution based on gender and UVA. Statistically significant differences were individuated for each eye, separately (RE: *p* = 0.032; LE: *p* = 0.015).

However, significant differences were individuated between gender and UVA in each eye, separately (RE: *p* = 0.032; LE: *p* = 0.015, Figure 4).

Among the patients enrolled, 74 (15%) underwent their last ophthalmological examination within the last year; a total of 298 (60%) had their eyes checked one year ago or before and 122 (25%) had never had ophthalmological consultations. A total of 391 patients (79%) used to wear glasses before 2019, while 105 (21%) needed glasses and 34 (7%) required a change in their previous prescription since the pandemic ended. The vast majority of children and adolescents (436–87.9%) had continuous online classes during the pandemic period, while 348 (70.2%) reported spending more than 8 h every day in sedentary activities. A substantial daily increase in electronic device screen exposure was declared by 426 patients (87.6%), with most of them (253 patients, 51%) using preferentially a laptop. Indeed, 51 patients (10.4%) declared to have used video terminals for longer than 6 h a day, whereas 216 (43.6%) used these for between 5 and 6 h, 132 (26.6%) for between 2 and 4 h, and 96 (19.4%) for less than 2 h per day. More than half (50.8%) of the screened patients reported the habit of watching electronic displays in conditions of scarce luminosity or darkness. A total of 254 (51.3%) patients declared to use electronic devices just for entertainment, 88 (17.7%) for work/study-related issues and 154 (31%) for both reasons.

## 4. Discussion

In this study, a large pediatric cohort of Italian individuals was screened to evaluate the prevalence of refractive errors within the school-aged population after the confinement measures adopted to limit the spread of the recent COVID-19 pandemic.

Our findings provide significant evidence that may be useful to understand the impact of the implemented restrictions as well as updated insights for the prevention of refractive errors in the context of the dramatic global shift to online learning for children and adolescents that took place throughout this period.

To contain the spread of the COVID-19 virus, consistent with the World Health Organization’s intentions, school-aged individuals all over the world forcefully stayed at home, did not attend school in person, and heavily limited almost every outdoor activity [20]. Indeed, many children switched to online classes, overexposing themselves to electronic screens (e.g., tablets, computers, smartphones) for educational and recreational reasons, as well as to socially engage with peers [21].

The unhealthy behaviors we outlined in our questionnaire far exceeded the previously reported data in the published literature [22]. Recent studies have reported that the excessive exposure to electronic screens significantly overstimulates accommodative effort induced by the close-working distance and, thus, represents a huge risk factor for inducing refractive errors in school-aged children [23]. Indeed, a pre-pandemic epidemiological study found that only 34.6% of children and adolescents reported spending more than 2 h per day in front of a screen [24]. After the pandemic, 80.9% of the children and adolescents we evaluated tend to spend more than 2 h/day looking at electronic screens. Even though the genes responsible for myopia are thought to be characterized by low expressivity and penetrance, the surrounding environment seems to play a pivotal role in their expression. A recent study conducted on 3831 adolescents confirmed that every 1 h increase in daily digital screen exposure is associated with a significant increase of 1.26 in the odds ratio (OR) (95% Confidence Interval (CI): 1.21–1.31, *p* < 0.001]), corresponding to higher risks of myopic progression. In addition, the use of computers (OR: 1.813, 95% CI: 1.05–3.12, *p* = 0.032) and smartphones (OR: 2.02, 95% CI: 1.19–3.43, *p* = 0.009) were demonstrated to be linked to higher risks of myopic progression. Conversely, practicing outdoor exercise four to six times per week (OR: 0.745, 95% CI: 0.568–0.977; *p* = 0.034) and one to three times per week (OR: 0.829, 95% CI: 0.686–0.991; *p* = 0.048) were displayed to be associated with lower risks of myopia progression [25].

On a physiological level, near-vision application has been described to induce ciliary muscles’ thickening and was also found to be significantly associated with increased refractive power of the retina, prolonged eye axis, and, in general, myopic vision disorder [26,27]. Under this perspective, the adolescent eye, which is not yet fully developed, and the over-elicited near-vision stimulation from electronic displays may eventually lead to an over-extended hyperopic defocus exposure. A recent study has indeed proposed that chronic hyperopic defocus may trigger a compensating myopic axial eye increase, which prematurely affects refractive vision development in animal models [28].

Epidemiological characterization of digital screen use effects has also been conducted, suggesting it is a leading risk factor of visual disorder onset among children [29]. Indeed, it highlights that eye strain and fatigue symptoms, classically classified within asthenopic symptoms, are typically reported to occur after as little as 60 min of smartphone use, and that the duration of computer use among children is significantly and closely associated with refractive error progression [29,30].

In a study conducted on Danish individuals, the authors reported that digital screen use accounts for about 25% of the observed prevalence of myopia, with an increased myopic risk if digital screens were used for longer than 6 h per day [25]. Saxena et al., instead, reported that screen time exposure for longer than 2 h each day positively correlated with rates of myopic progression, and the effect of screen time was even more prominent in areas where myopia prevalence was reported to be low [31]. All these abovementioned findings, confirmed also by our report, support the current idea that home confinement indeed played a major causative role in the prevalence increase of adolescents’ refractive errors at the end of the pandemic period. In fact, in comparison with the pre-COVID era, our findings confirm the trend also described in other published reports with regards to both myopic and hyperopic refractive errors [32,33]. Indeed, previous meta-analyses reported that, before 2019, the age-matched estimated pool prevalence of myopia and hyperopia were 11.7% and 4.6%, respectively [32].

Our cohort displayed a significantly higher trend after the pandemic, as 14.6% of patients were diagnosed with myopia/myopic astigmatism and 10.5% with hyperopia/hyperopic astigmatism. The same finding was also obtained with regards to the mean spherical equivalent (−0.06 ± 1.44 diopters), which a previous retrospective observational study found is significantly (*p* = 0.005) reduced in comparison to the pre-pandemic reports [33]. In particular, the authors evaluated 883 children, aged between 5 and 12 years old, and reported a mean spherical equivalent of −0.08 ± 1.44 diopters, which showed a significant mean reduction compared with the referred-to epidemiological data obtained two years prior to the pandemic outbreak (0.35 ± 1.75 diopters in 2019 and 0.34 ± 1.41 diopters in 2018) [33].

Our study may have several implications as it could help to guide prevention and future visual-disability control strategies. These may include policies that could support comprehensive measures to lessen the time spent learning online, overall reducing electronic devices overuse, and to increase outdoor engagement through regular physical activity at school and in the community [34]. In addition, another finding of utmost importance that resulted from the screening questionnaire is that 85% of our cohort did not undergo ophthalmologic examinations regularly and 25% had not been assessed even once by a physician. The resulting effects of a substantial lack of self-care regarding eye health would explain why almost one-fifth of our cohort had an undiagnosed, uncorrected, refractive error with a consequent quality of life impairment. Raising awareness and promoting conscious eyecare and education would be pivotal in the management of the burden of next generation visual impairment. It would be very important, for overall society, to spread the message that regular eye visits in childhood are crucial to have a safe development of visual function. Late ophthalmic checks, in fact, could lead to an eventual amblyopia diagnosis that could lead to a lifetime of monocular visual impairment. This would be both an individual problem, because the subjects with amblyopia will have difficulties in reaching a high level of education and/or work despite having all the other qualities to get them. This would also be a collective problem because amblyopia subjects would have difficulties in obtaining a satisfactory social role, leading the health system or other welfare administrations to take care of them.

Our study had several limitations. First, myopia was evaluated by combining the retro illuminated decimal Snellen chart and the noncycloplegic autorefraction followed by subjective refraction to determine the refractive error. Deducting refractive errors in the absence of cycloplegia may have overestimated myopic powers and underestimated hyperopic powers, especially considering the young age range of our cohort [35,36]. Since the RE measurement with cycloplegia would not be adequate for large population studies such as ours, we suggest that these findings would be consistent with a positive screening for RE presence, rather than a proper diagnosis. Previous studies have also demonstrated that the diagnostic methods we adopted in our screening reached sensitivity and specificity of 92.4% and 89.5%, respectively, in large population surveys [37]. Thus, although not adequate for RE diagnosis in a clinical setting, we consider this method as an acceptable compromise for large population surveys. Secondly, the study design and the limited setting-related clinical resources did not allow a proper follow-up, restricting access to further significant clinical investigations and imaging data that could have been useful to evaluate. In addition, even though the lockdown-related restrictions unequivocally had a major impact on the variables investigated in our study, it is also important to take into account that the measurements were conducted in 2022, about two years later the strictest measured were adopted. This relatively long period elapsed between the two events should be taken into consideration as it may have affected the effects of the quarantine. Finally, it is important to highlight that the results demonstrated in the current study represent a specific, although very populous, region in Italy, therefore limiting their generalizability to the other regions of the same country.

## 5. Conclusions

In conclusion, according to the data obtained in our cohort, pediatric patients ranging between 6 and 11 years of age appear to have a higher prevalence of refractive errors than before. This finding could be associated with COVID-19 pandemic restrictions, applied to reduce the spread of the disease in recent years. Considering the results observed, an ophthalmologic evaluation within the school age appears to be crucial to timely diagnose refractive errors. Of utmost importance should also be adopting measures to avoid the consolidation of all the aforementioned unhealthy behaviors among children.

Given the fact that the widespread prevalence of visual impairments among youths has been growing at an alarming rate over last few years, it is therefore a rising serious global public health concern, and governments and health systems worldwide should raise public awareness regarding the importance of undergoing routine ophthalmological visits to timely detect and correct visual conditions. Appropriate media campaigns of sensibilization should be promoted and medical structures availability and accessibility should be enhanced to allow easier access to eyecare. Without such implementations, an even sharper rise in the prevalence of visual disabilities might be observed in the near future.

For these reasons, further randomized, prospective studies on this wide topic, with longer follow-ups and larger sample sizes are warranted to have a more accurate evaluation of such serious problems, to better characterize and assess potential predictors of increased risk in the incidence of refractive errors and to achieve overall better preventive management of such conditions.

## Data Availability

Data presented in the manuscript are available from the corresponding authors upon reasonable request.
